# Flowering Phenological Patterns of Entomophilous Plants in an Alpine Grassland of the Qilian Mountains

**DOI:** 10.3390/plants15131973

**Published:** 2026-06-26

**Authors:** Wen Wang, Heng Ren, Jun Du, Zhibin He, Chenxin Miao, Juanjuan Wu, Dengke Ma

**Affiliations:** 1 Key Laboratory of Ecological Safety and Sustainable Development in Arid Lands, Northwest Institute of Eco-Environment and Resources, Chinese Academy of Sciences, Lanzhou 730000, China; 2 Key Laboratory of Knowledge Computing and Intelligent Decision, Lanzhou 730000, China; 3 Linze Inland River Basin Research Station, Northwest Institute of Eco-Environment and Resources, Chinese Academy of Sciences, Lanzhou 730000, China

**Keywords:** flowering phenology, alpine grassland, temporal niche, functional group, mass flowering, Qilian Mountains

## Abstract

Flowering phenology is a critical life-history trait determining the reproductive success of entomophilous plants in alpine ecosystems. However, comprehensive characterizations and cross-level comparative studies of these phenological patterns remains limited. In this study, we conducted high-frequency phenological monitoring of 36 entomophilous species across 15 families in a typical alpine grassland in the Qilian Mountains, extracting parameters at the individual, population, functional group, and community levels. Our results demonstrate that the community flowering season lasted approximately 100 days, beginning in early June and terminating in early September. Community flower abundance exhibited a bimodal distribution, reaching distinct peaks in early July and early August. Overall flowering synchrony among plants was generally low, indicating clear temporal niche differentiation. The plant community comprised early-, mid-, and late-flowering groups. The temporal overlap of early- and mid-flowering species jointly contributed to the first floral peak in early July, whereas late-flowering species drove the second peak in early August. Among these functional groups, the early-flowering group accounted for the lowest proportion of species, whereas the late-flowering group accounted for the highest. Both early- and late-flowering groups tended toward mass-flowering strategies, while the mid-flowering group leaned toward steady-flowering. At the interspecific level, earlier-flowering species typically exhibited shorter flowering durations and more right-skewed floral distributions, meaning the interval from the first to the peak flowering date was shorter than that from the peak to the last flowering date. At the intraspecific level, individuals that initiated flowering early or late typically exhibited longer flowering durations but lower synchrony with conspecifics; conversely, individuals blooming during the middle of the population’s season exhibited the opposite pattern. These findings systematically delineate the hierarchical temporal structure of alpine entomophilous communities, objectively reflecting the differential utilization of a limited growing season through inter- and intraspecific phenological variation.

## 1. Introduction

Flowering phenology, a crucial rhythmic event in the sexual reproduction of entomophilous plants, is widely recognized as a primary determinant of fitness for many angiosperms [1,2]. These plants fundamentally rely on animal vectors for pollen transfer and fertilization, typically utilizing conspicuous floral displays, scent, and nectar to attract pollinators. Such mutualistic interactions make their reproduction highly sensitive to climate-induced mismatches [3]. The onset of flowering marks the critical transition from vegetative to reproductive growth [4]. This shift necessitates the reallocation of resources and reflects an evolutionary trade-off of limited assets (such as nutrients, energy, and time) across developmental stages [5,6]. Furthermore, phenological traits, including flowering duration and synchrony, determine reproductive success by defining the window for pollinator visitation and the timing of fruit set [2]. Premature flowering, coupled with insufficient vegetative growth, yields underdeveloped plants lacking the resource reserves required for fruit maturation; conversely, delayed flowering shortens the reproductive period, preventing full fruit development [7]. Low intraspecific flowering synchrony can also impede outcrossing [3]. Shifts in flowering times can directly reduce plant fitness by causing phenological mismatches with pollinators [8,9]. Simultaneously, such mismatches deplete available foraging resources, reducing pollinator fitness [10,11], which consequently limits the pollination services these plants can acquire, indirectly compromising their reproduction success [12].

Driven by both genetic and environmental factors, flowering phenology exhibits distinct variation across different ecological scales [1]. Intraspecific phenological patterns reflect diverse flowering modes and reproductive strategies among individuals [13]. Constrained by short growing seasons, most plants in cold and temperate ecosystems adopt a mass-flowering strategy. These plants maximize their peak floral display by producing an abundance of flowers over a brief period, thereby maintaining high intraspecific synchrony [1,14]. Conversely, other species exhibit a steady-flowering pattern, buffering against reproductive risks by extending their flowering duration and exhibiting lower intraspecific synchrony [13]. Interspecific differences in flowering phenology reflect the partitioning of reproductive temporal niches, directly influencing species coexistence and competition [3,15,16], while flowering synchrony among sympatric species relying on shared pollinators modulates the balance between competitive and facilitative interactions for pollinator attraction [15,17,18].

Under the combined selective pressures of abiotic and biotic factors, plant species frequently form distinct flowering functional groups through the aggregation or differentiation of temporal niches [16,19,20,21]. Consequently, their phenological patterns reflect specific adaptive trade-offs [22,23]. Specifically, while early-flowering species benefit from ample time for seed development, they face heightened risks from environmental stressors, such as early-season frost, and have restricted periods for vegetative resource accumulation [24,25]. Mid-flowering species experience optimal conditions and pollinator availability but must contend with intensified competition for essential resources and pollinators during the peak community blooming period [26]. Conversely, late-flowering species benefit from an extended period of vegetative growth but encounter risks such as end-of-season resource depletion and potential reproductive failure due to the abrupt termination of the growing season [23]. In addition, floral distribution dynamics at the community level are not a simple aggregation of individual taxonomic traits; rather, they are jointly driven by the phenological characteristics and relative abundances of different functional groups [18]. Therefore, elucidating these hierarchical phenological patterns is crucial for understanding the structural stability and evolutionary trajectories of plant communities.

As a critical vegetation type in alpine ecosystems, alpine grasslands are characterized by short growing seasons, substantial diurnal temperature fluctuations, high precipitation seasonality, and nutrient-poor soils [20,24]. Plants inhabiting these regions must complete their reproductive cycles within narrow temporal windows, rendering flowering phenology a vital adaptive trait. However, due to the inherent challenges of multi-species and multi-scale monitoring, systematic characterizations of phenological patterns across entire entomophilous plant communities remain scarce. To address this gap, we investigated 36 entomophilous species in a typical alpine grassland (2931 m a.s.l.) in the Qilian Mountains of the northeastern Qinghai-Tibetan Plateau. Through high-frequency phenological monitoring over the growing season, we extracted a comprehensive suite of parameters, including first, peak, and last flowering date, together with flowering duration, synchrony, and curve skewness. We aimed to explore phenological patterns, flowering mode characteristics, and the associations among these parameters by integrating the individual, population, and functional group levels. These findings will provide a more comprehensive foundational understanding of phenological processes in natural alpine communities and offer a scientific basis for predicting flowering phenology and subsequent community reassembly under climate change scenarios [21,27].

## 2. Results

### 2.1. Flowering Phenology Patterns at Intra-Specific Individual Level

At the intraspecific individual level, the flowering phenological characteristics of different entomophilous plant species exhibited significant variation. The median first, peak, and last flowering dates (DOY) of monitored individuals across species ranged from 159–218, 160–226, and 162–242, respectively, with substantial variation in amplitude (Table 1). For instance, the variation ranges for species such as *Aster souliei* and *Gentiana pseudoaquatica* exceeded 50 days, whereas those for *Bistorta vivipara* and *Aster lautureanus* were less than 5 days. The median individual flowering duration across species ranged from 1 to 33 days (Table 1), with *Taraxacum mongolicum* and *G. pseudoaquatica* exhibiting the shortest durations (1 days), and *Braya humilis* the longest (33 days). The flowering synchrony index (*S_i_*) ranged from 0.10 to 0.97 (Table 1); *T. mongolicum* and *G. pseudoaquatica* had the lowest values, while *A. lautureanus* and *B. vivipara* showed relatively high values exceeding 0.9.

**Figure 1:**
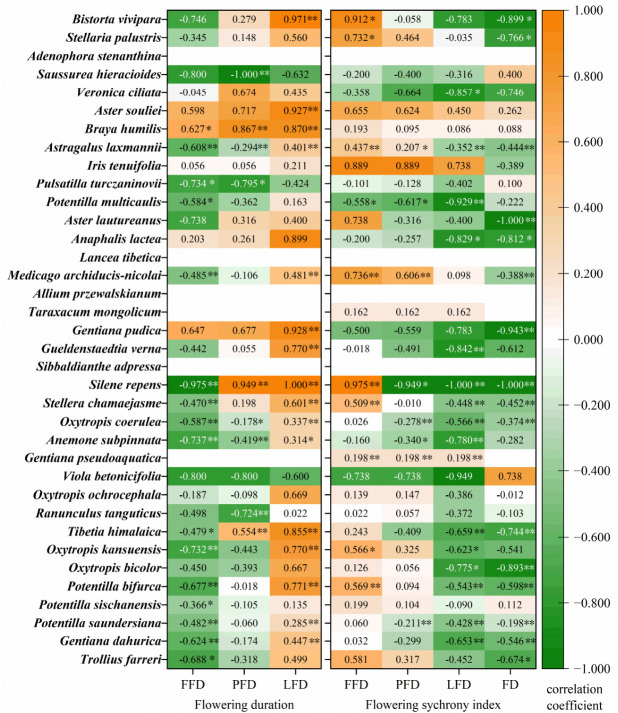
Correlation between individual flowering parameters. The correlation coefficient shown in the figure is Spearman correlation coefficient. FFD: first flowering date; PFD: peak flowering date; LFD: last flowering date; FD: flowering duration. * indicates a significant correlation (*p* < 0.05), and ** indicates a highly significant correlation (*p* < 0.01).

Intraspecific correlation analysis of these parameters revealed that among the 32 plant species analyzed, individual flowering duration in the majority (23 species) was negatively correlated with the first flowering date and positively correlated with the last flowering date. Among these, 11 species showed significant correlations with both first and last flowering dates (*p* < 0.05), 3 species were significantly correlated only with the first flowering date (*p* < 0.05), and 1 species was significantly correlated only with the last flowering date (*p* < 0.05) (Figure 1). This indicates that for most species, individuals that initiate flowering earlier and terminate flowering later possess a longer flowering duration. Furthermore, in 22 species, individual flowering synchrony was negatively correlated with both the last flowering date and flowering duration, with 10 of these species showing significant correlations with both parameters (*p* < 0.05) (Figure 1). This suggests that, for most plants, early-flowering individuals with extended flowering durations and late-flowering individuals with delayed offset dates exhibit relatively lower flowering synchrony. In contrast, individuals blooming during the intermediate phase of the population’s flowering period display higher synchrony.

### 2.2. Flowering Phenological Patterns at Population Level

**Figure 2:**
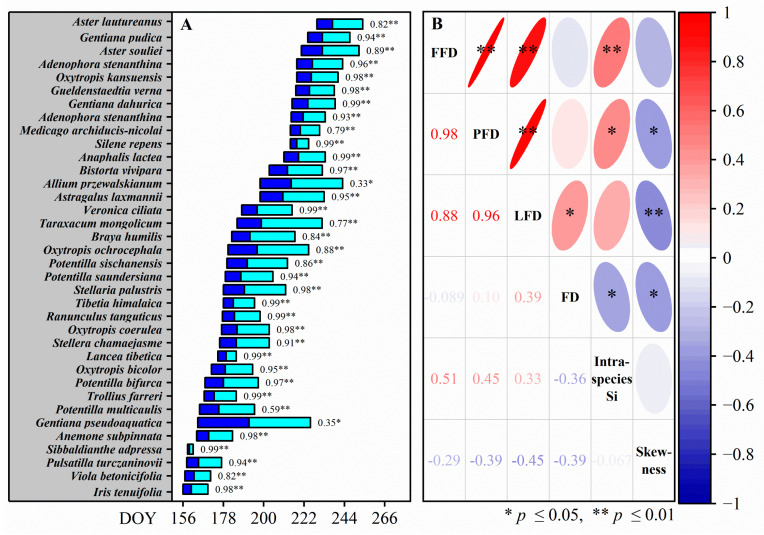
Flowering phenological characteristic at population level. (**A**). Flowering phenological characteristics at the population level of plants. The starting point of the dark blue band is the first flowering time at species level of each plant, the intersection of the two color bands is the peak flowering time, and the end point of the light blue band is the end flowering time. (**B**). Correlation among different flowering parameters at the population level. The correlation coefficient shown in the figure is Pearson’s correlation coefficient. FFD: first flowering date; PFD: peak flowering date; LFD: last flowering date; FD: flowering duration; Intraspecies Si: flowering synchrony index among individuals within each species. The numbers adjacent to the bars in Panel A indicate the coefficient of determination (*R*^2^) for the Gumbel model fitting of the flowering period for each species, with asterisks denoting the level of statistical significance (* *p* < 0.05, ** *p* < 0.01).

**Figure 3:**
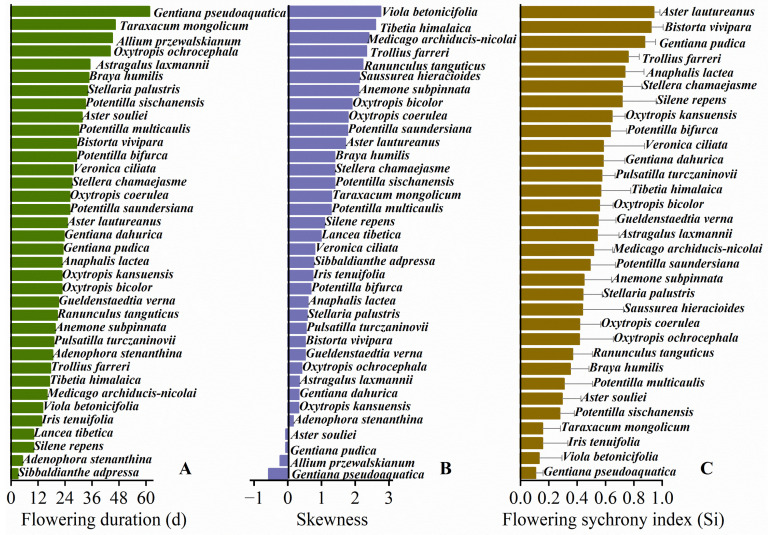
Flowering phenological parameters of different plant species. (**A**). Flowering duration at the population level of plants. (**B**). Skewness of flower abundance distribution at population of plants. (**C**). Intraspecific flowering synchrony index of plants. A positive skewness value (>0) indicates a right-skewed distribution, meaning the duration from the onset of flowering to the peak is shorter than the duration from the peak to the end of flowering. The mean value of the flowering synchrony index of different individuals within each plant species, with an error bar of one standard deviation, is shown in figure.

At the population level, the flower abundance of all 36 entomophilous plant species exhibited a unimodal distribution over time (Figure 2A). From June 5th (DOY: 156), when *Iris tenuifolia* initiated flowering, to September 9th (DOY: 252), when *A. lautureanus* reached the end of its flowering period, other plant species sequentially progressed through various phenological stages (Figure 2A). This indicates that the flowering periods of different entomophilous plants in the alpine grassland span the entire reproductive growing season, with phenological overlap occurring among species. The average population flowering duration across species was 25 ± 12 days, yet it exhibited substantial interspecific variation. The longest flowering duration (*G. pseudoaquatica*, 62 days) was approximately 20 times that of the shortest (*Sibbaldianthe adpressa*, 3 days) (Figure 3A). The majority of species (32 species, accounting for 89%) had skewness values ranging from 0.16 to 2.77, displaying a significant right-skewed distribution (Figure 3B). This indicates a rapid progression to peak flowering following onset, followed by a slower decline towards the end of the flowering period. Concurrently, most species (25 species, 78%) had intraspecific flowering synchrony indices between 0.2 and 0.8 (Figure 3C), representing moderately low to moderately high synchrony.

Regarding the correlations among population-level flowering parameters, highly significant positive correlations (correlation coefficient > 0.9) were observed among the first, peak, and last flowering dates (Figure 2B), demonstrating a high degree of intrinsic coordination among these three phenological events at the population level. However, flowering duration was significantly positively correlated only with the last flowering date (*p* < 0.05) (Figure 2B), suggesting that the length of specific species’ flowering period is largely determined by its termination date. Intraspecific flowering synchrony was positively correlated with the first, peak, and last flowering dates, showing highly significant (*p* < 0.01) and significant (*p* < 0.05) positive correlations with the first and peak flowering dates, respectively. Conversely, skewness was negatively correlated with the first, peak, and last flowering dates, exhibiting highly significant negative correlations with the peak (*p* < 0.01) and last (*p* < 0.05) flowering dates (Figure 2B). This indicates that species with earlier flowering times exhibit lower intraspecific flowering synchrony and a more right-skewed floral distribution. Furthermore, flowering synchrony is more strongly influenced by the first flowering date (correlation coefficient = 0.51), whereas skewness is more strongly influenced by the last flowering date (correlation coefficient = −0.45) (Figure 2B). In addition, both intraspecific flowering synchrony and skewness were significantly negatively correlated with flowering duration (*p* < 0.05) (Figure 2B), indicating that species with shorter flowering periods possess higher intraspecific flowering synchrony and a more strongly right-skewed floral distribution.

### 2.3. Flowering Phenological Patterns at Functional Group Level

**Figure 4:**
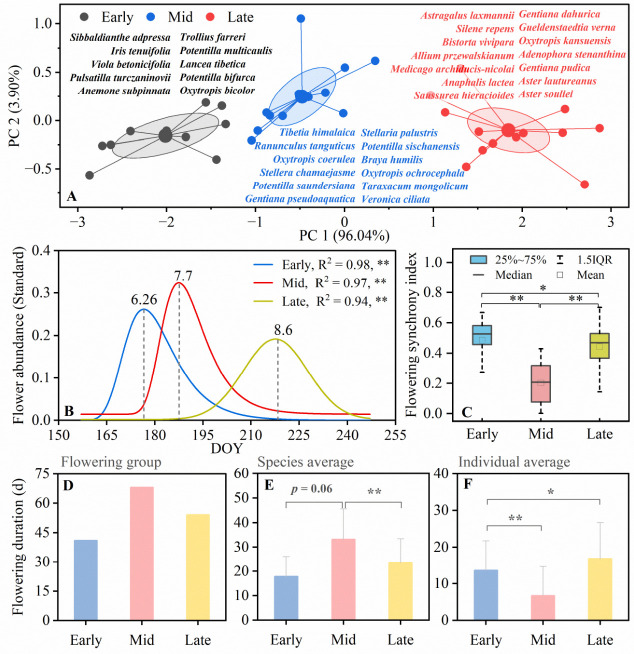
Flowering phenological characteristic at functional group level. (**A**). Division of functional groups of early-, mid- and late-flowering plants. The K-means clustering results based on the first flowering time, the peak flowering time, and the end flowering time of each species and the species composition of the early-flowering, mid-flowering and late-flowering plant groups. (**B**). Temporal dynamics of flower abundance of different plant groups. (**C**). Flowering synchrony index of different plant groups. (**D**). Total flowering duration at the functional group level, representing the effective duration estimated from the aggregated flower abundance curve of the entire group. (**E**). Mean flowering duration at the species-population level, displayed as the mean ± one standard deviation of the population-level durations for all species within each group. (**F**). Mean flowering duration at the individual level, shown as the mean ± one standard deviation of the individual-level durations across all plants within each group. * indicates significant difference (*p* < 0.05), ** indicates very significant difference (*p* < 0.01).

K-means clustering analysis, based on the parameters of the first, peak, and last flowering dates, divided the community into three functional groups: early-flowering, mid-flowering, and late-flowering. Statistical tests revealed highly significant differences in the first, peak, and last flowering dates among these groups (*p* < 0.01). The late-flowering group contained the highest number of plant species (14 species), followed by the mid-flowering group (12 species) and the early-flowering group (10 species) (Figure 4A). The calculated importance values for the early- and mid-flowering groups were 21.1% and 26.6%, respectively, whereas the late-flowering group exhibited the highest dominance at 52.3%, which is approximately twice that of the other two groups. This indicates that the late-flowering group occupies a more critical position within the entomophilous community.

Within each functional group, flower abundance exhibited a unimodal distribution over time (Figure 4B). The peak flowering dates of the early- and mid-flowering groups were relatively close, separated by only 11 days (22 June to 7 July). In contrast, the peaks of the mid- and late-flowering groups were separated by 30 days (7 July to 6 August), an interval more than twice as long as that between the early and mid-flowering peaks (Figure 4B). The flowering periods spanned from 5 June to 16 July for the early-flowering group, 13 June to 20 August for the mid-flowering group, and 17 July to 9 September for the late-flowering group (Figure 4B). It is evident that the flowering periods of the three groups overlapped. Furthermore, at the functional group level, the mid-flowering group possessed the longest flowering duration, followed by the late-flowering group, with the early-flowering group being the shortest (Figure 4D). Additionally, the species mean flowering duration of the mid-flowering group was significantly longer than that of the late-flowering group (*p* < 0.01) and marginally significantly longer than that of the early-flowering group (*p* = 0.06) (Figure 4E). Conversely, its individual mean flowering duration was significantly the shortest among the three groups (*p* < 0.05). Considering that the mid-flowering group also exhibited significantly the lowest individual flowering synchrony (*p* < 0.05) (Figure 4C), it is apparent that its individual flowering times are the most dispersed, whereas the individual flowering times of the early- and late-flowering plants are more concentrated.

### 2.4. Phenological Patterns at the Community Level

**Figure 5:**
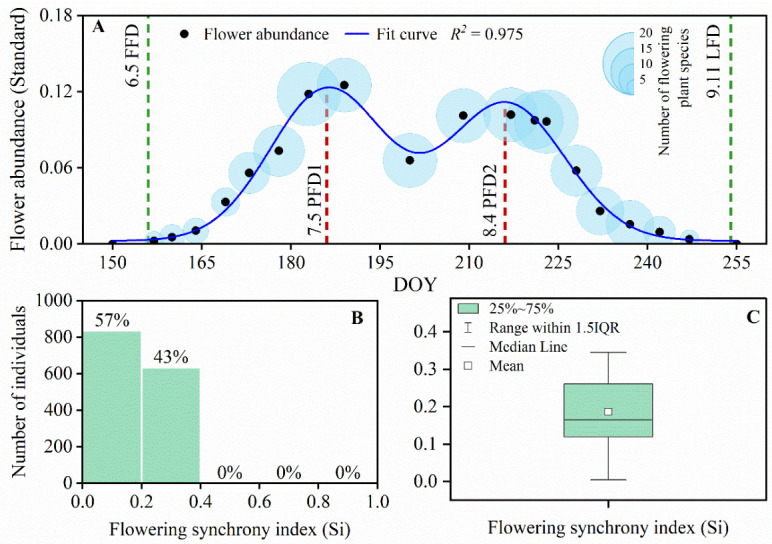
Flowering phenological characteristic at community level. (**A**). Temporal dynamic of flower abundance and flowering species in plant community. (**B**). Distribution of flowering synchrony index of all monitoring individuals. (**C**). Flowering synchrony index of plant community.

At the community level, the flowering period of entomophilous plants extended from early June to early September, lasting a total of 98 days (Figure 5A). Community flower abundance exhibited a pronounced bimodal distribution over time, with two distinct peaks occurring in early July and early August. The flower abundance of the first peak was slightly higher than that of the second (Figure 5A). Notably, the periods with the maximum number of flowering species preceded the first abundance peak and followed the second, while remaining at a relatively high level between these two peaks (Figure 5A). Concurrently, based on the mean flowering synchrony of all monitored individuals within the community, individual synchrony ranged from 0 to 0.35, with up to 57% of individuals falling between 0 and 0.2 (Figure 5B). The overall mean was exceptionally low at 0.19 ± 0.08 (Figure 5C), indicating that the flowering times of different individual plants in the natural community are highly dispersed throughout the entire reproductive growing season.

## 3. Discussion

### 3.1. Temporal Niche Differentiation Shapes Community Flowering Phenology

In alpine grassland ecosystems, plant growth and development are strictly constrained by persistent low temperatures, short growing season, and limited resources availability [12,20,24]. This study found that the flowering period of entomophilous plants in this region typically extends from early June to early September, during which favorable hydrothermal conditions provide a necessary bioclimatic window for plant reproduction and insect activities. Due to the restricted reproductive time, species exhibit divergence or convergence in their flowering phenology through the partitioning of or competition for reproductive timing [26,28]. Flowering periods of various species at the study site span the entire summer, while the low flowering synchrony among all individuals in the community indicates that the degree of overlap is relatively low, reflecting clear temporal niche differentiation [16].

The temporal distribution of flowering exhibiting distinct clustering that forms three functional groups. Early- and mid-flowering species concentrate their peak flowering in the early stage (mid-June to mid-July), whereas late-flowering species peak in the late stage (late July to late August). Consequently, community flower abundance exhibits a bimodal distribution, with the peak in the early stage being higher. This indicates that in alpine grasslands, a larger proportion of species (early- and mid-flowering plants) tend to flower early rather than deferring reproduction—a pattern consistent with findings from Mount Everest [24], which is likely driven by the evolutionary advantage of avoiding intense interspecific competition for pollinators later in the season [26]. Such temporal aggregation and differentiation likely evolved under genetic control and long-term environmental adaptation [22,23].

Furthermore, under ongoing climate warming, the phenological responses of different species and phenological parameters often lack synchronization [25], leading to diverse shifts that alter interspecific co-flowering patterns, reproductive season duration, and community floral abundance distributions [18,25]. In this study, significant interspecific differences in both population- and individual-level flowering characteristics were observed, alongside varying relative abundances among species and functional groups. Therefore, it is inferred that future changes in species-specific population sizes and flowering phenology will differentially impact community-level patterns, further exacerbating the complexity of overall phenological shifts [16,21]. This reinforces the concept that community floral distribution is not a mere aggregation of species traits, but rather a dynamic pattern jointly driven by the phenological rhythms and relative abundances of its constituents [18]. Consequently, multi-scale and multi-parameter assessments are essential for accurately predicting how climate-induced phenological shifts will reshape alpine ecological structures.

### 3.2. Divergent Flowering Strategies Across Functional Groups

To adapt to specific habitats, plants evolve distinct flowering strategies within their temporal niches. These strategies generally fall along a continuum between “mass-flowering” and “steady-flowering” patterns. Constrained by short growing seasons, most cold- and temperate-ecosystem species lean toward mass-flowering [1,14]. In this study, the population-level flowering duration and synchrony of entomophilous plants exhibited a continuous distribution, indicating that their flowering patterns span this same continuum. Furthermore, population flowering duration was significantly negatively correlated with flowering synchrony across species. Notably, *A. lautureanus* and *G. pseudoaquatica* exemplified mass- and steady-flowering patterns, respectively. The former exhibited nine times the synchrony of the latter but only half the population duration, underscoring the profound differentiation between these distinct strategies) [1]. Moreover, plants within the same family flowering at different times displayed contrasting patterns. For example, among Gentianaceae annuals, the mid-flowering *G. pseudoaquatica* possessed a long flowering duration and low synchrony, showing a clear tendency toward steady-flowering, whereas the late-flowering *Gentiana pudica* exhibited the opposite pattern. Similarly, among Asteraceae perennials, the mid-flowering *T. mongolicum* leaned toward steady-flowering, while the late-flowering *A. lautureanus* tended toward mass-flowering. This suggests that interspecific variation in flowering strategies may be primarily determined by flowering timing.

At the functional group level, early-flowering species exhibited higher individual synchrony and longer individual flowering durations, but shorter population-level flowering durations. Mid-flowering species displayed the inverse patterns, while late-flowering species exhibited intermediate characteristics. These differences suggest that early-flowering species concentrate their reproductive efforts into a brief window, mid-flowering species prolong their duration for steady-state flowering, and late-flowering species adopt a transitional strategy. The early-flowering group is characterized by fewer species, dispersed interspecific peak flowering dates, and relatively low community flower abundance. These factors likely result in smaller group-level floral displays and, consequently, weaker pollinator attraction, given the generally low pollinator abundances early in the season [26]. Under such conditions, adopting a mass-flowering strategy enables these species to enhance their respective population-level floral displays, thereby attracting pollinators more effectively [29,30]. Conversely, mid-flowering species bloom during peak community floral abundance and face intense interspecific competition for pollinators due to concentrated peak flowering dates. By adopting an extended, steady-flowering strategy, these species can disperse competitive pressure, thereby increasing the probability of insect visitation and outcrossing for the entire population.

For late-flowering species, both population flowering duration and synchrony are intermediate, reflecting a strategic reproductive trade-off. Late-season low temperatures restrict both floral maintenance and pollinator activity. Consequently, constrained by limited reproductive time, late-flowering species must bloom with relatively high synchrony to complete reproduction before the growing season ends [23]. Simultaneously, they extend their flowering duration to physiological limits to maximize pollinator visits, thereby buffering against the dual risks of late-season cold stress and competition from lingering mid-flowering species [2,27]. This highlights a fundamental divergence in selective pressures: while the flowering strategies of early- and mid-flowering species are predominantly shaped by biotic factors such as the pollination environment, those of late-flowering species are primarily constrained by abiotic factors such as available reproductive time [12,16].

### 3.3. Interrelations and Temporal Dynamics of Phenological Parameters

Influenced by internal traits and environmental factors, population flowering periods occupy distinct temporal niches and exhibit individual variation [1,31]. In this study, highly significant positive correlations among the first, peak, and last flowering dates indicate that subsequent phenological events are temporally constrained by preceding ones. Furthermore, population flowering duration was significantly positively correlated only with the last flowering date, and the early-flowering group exhibited the shortest average duration. This suggests that early-flowering entomophilous plants possess shorter population flowering periods, reflecting a mass-flowering strategy to concentrate floral production. While a previous alpine meadow study reported longer durations for early-flowering species [32], this discrepancy is likely attributable to their inclusion of wind-pollinated grasses, which typically flower very early in alpine ecosystems [20]. Indeed, entomophilous plants in our community bloomed slightly later than certain Poaceae species, suggesting they possess distinct phenological patterns compared to wind-pollinated plants.

At the individual level, floral longevity—the duration a flower remains functional—is a critical trait exhibiting substantial inter- and intraspecific variation [33,34]. For instance, *T. mongolicum* and *G. pseudoaquatica* exhibit ephemeral blooms, whereas the individual flowering duration of *Braya humilis* exceeded 30 days. We found that for most species, individual flowering duration was negatively correlated with both first and last flowering dates while individual flowering synchrony was positively correlated with the last flowering date and negatively correlated with duration. This implies that individuals blooming near the fringes of the population’s season exhibit extended floral longevity and lower synchrony, whereas those blooming during the peak display shorter longevity and higher synchrony. A plausible explanation is that massive floral displays during the peak facilitate insect visitation [35], enabling prompt pollination and fruiting [36,37]. Conversely, lower floral abundance at the seasonal fringes attracts fewer pollinators [26]; thus, plants likely extend their floral longevity to maximize pollination probability [38,39]. Consequently, intraspecific pollination environments are highly variable, where pollination probability may act as a primary driver of floral longevity [40].

In grassland and montane ecosystems, intraspecific floral distribution is typically right-skewed, characterized by a rapid peak and gradual decline [41,42]. Consistent with these patterns, the population-level flower abundance of most entomophilous plants in this study also exhibited varying degrees of right-skewness. This pattern arises because flowering onset responds synchronously to specific environmental cues, whereas termination is influenced by more diverse and stochastic factors, leading to greater temporal dispersion [43]. Additionally, larger individuals with superior nutrient reserves tend to flower earlier and exhibit higher seed-setting capabilities under sufficient pollination [16,26]. The preferential allocation of intraspecific resources to these early-flowering individuals further exacerbates the right-skewness [44,45]. Such selective pressures favoring early reproduction likely drive this right-skewed phenology [46]. Furthermore, we found that species-level skewness was significantly negatively correlated with peak and last flowering dates, indicating that earlier-flowering species exhibit more pronounced right-skewness, corroborating recent findings [47]. Simultaneously, skewness was significantly negatively correlated with population flowering duration, suggesting that the compressed flowering duration characteristic of early-flowering plants may be the underlying cause of their pronounced right-skewed distributions.

### 3.4. Study Limitations and Future Directions

While this study provides fine-scale insights into the hierarchical phenological patterns of alpine entomophilous plants, its scope is primarily focused on a single growing season within a specific plot. Although this localized approach enabled rigorous, high-frequency tracking of individual-level phenology, the lack of spatial replication and multi-year data may limit the broader generalizability of these responses, particularly given that alpine plant phenology is highly sensitive to landscape heterogeneity and interannual climatic fluctuations [16,27]. To build upon these foundational temporal patterns, it is crucial for future studies to expand the spatial and temporal monitoring scales while directly incorporating pollinator observation data. Integrating plant-pollinator interaction networks into the research framework will provide a more comprehensive understanding of plant-insect coupling mechanisms [12,28], ultimately facilitating a more accurate assessment of ecosystem functional resilience under global climate change [21].

## 4. Materials and Methods

### 4.1. Study Area

The study was conducted in a typical alpine grassland community located in the upper reaches of the Heihe River, within the middle section of the Qilian Mountain National Nature Reserve (2931 m a.s.l., 100°13′22″ E, 38°34′35″ N). This region is characterized by a temperate continental climate, strongly influenced by the atmospheric circulation of the Qinghai-Tibetan Plateau, resulting in an alpine semi-arid to semi-humid mountain forest-steppe climate. According to the 2022 meteorological data from a station located 5.5 km from the study site, the mean annual temperature was 2.5 °C, with average temperatures of −10.1 °C in winter and 14.1 °C in summer. The annual cumulative precipitation was approximately 350 mm, predominantly occurring between May and September. The plant growing season aligns with this precipitation pattern, lasting for over three months. Entomophilous herbaceous plants constitute approximately 50% of the target community, which features a high diversity of species. The study area serves as an autumn pasture and experiences moderate grazing during the late growing season. To eliminate disturbance from large livestock, the permanent monitoring plots and transects were enclosed with large-mesh fences before the onset of the growing season to prevent grazing and trampling by cattle and sheep.

### 4.2. Plot Setup and Plant Community Survey

**Figure 6:**
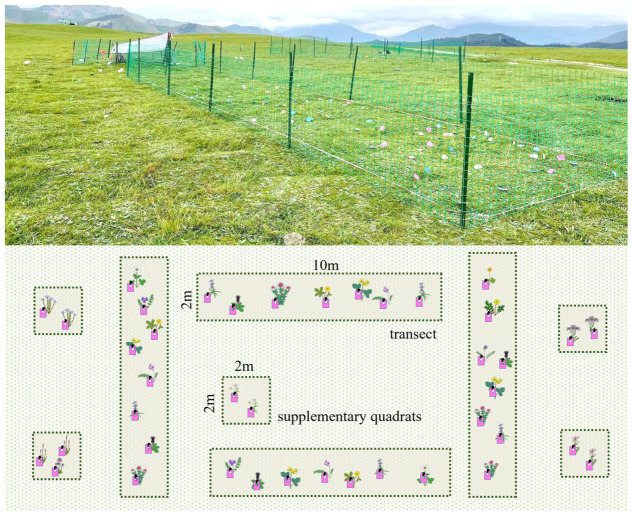
Diagram of plot layout.

Prior to the 2022 growing season, a 50 m × 50 m permanent plot was established at the study site as a long-term observation field for research on plant-pollinator interactions in alpine grasslands. Within this plot, four 2 m × 10 m transects were randomly deployed (Figure 6). Additionally, several 2 m × 2 m supplementary quadrats were established outside the transects for plant diversity surveys and flowering phenology monitoring (Figure 6). Simultaneously, two 1 m × 1 m herbaceous quadrats were randomly placed within each transect, and several small quadrats were set up around the periphery of the transects to conduct preliminary surveys of the entomophilous plant community composition. Based on the quadrat data, the importance value (IV) of each species was calculated as the average of its relative frequency, relative abundance, and relative coverage to evaluate its relative dominance. A total of 36 entomophilous plant species belonging to 15 families were recorded, and the community’s Simpson diversity index was 0.83 ± 0.05. Fabaceae, Asteraceae, and Rosaceae were the dominant taxonomic groups, collectively accounting for 61% of the total species. Species within the major families of these plants—such as Asteraceae, Rosaceae, Fabaceae, Ranunculaceae, and Brassicaceae—predominantly display mating systems characterized by self-incompatibility, non-spontaneous self-pollination, or limited selfing capacity, which typically rely heavily on insect pollinators for successful reproduction and seed set [48,49,50,51,52].

### 4.3. Flowering Phenology Monitoring

Flowering phenology monitoring targeted all entomophilous flowering plants present in the community. The monitoring period extended from May to September 2022, beginning with the opening of the first flower on the earliest individual and concluding with the fading of the last flower on the latest individual. Monitoring was conducted every three days, exclusively on clear days, and was postponed in the event of rain. For each entomophilous species found within the transects and supplementary quadrats, all flowering individuals were tagged to simultaneously acquire relative species abundance and flowering temporal niche information at both the individual and population levels (Figure 1). During each survey, individuals that flowered for the first time on that day were recorded and tagged; for already-tagged individuals, the number of open flowers was counted plant by plant until their flowering period ended. Through this approach, the entire monitoring process spanned a continuous duration of 109 days (Day of Year 147 to 255), completing a total of 20 valid survey rounds. This intensive sampling effort yielded continuous flowering observations and temporal dynamics of floral abundance for 1469 individuals across 36 plant species belonging to 15 families.

### 4.4. Phenological Parameter Extraction

Due to the phenological data are time-series data, all dates were converted to the day of the year (DOY, with 1 January as day 1) to facilitate statistical analysis. At the individual level, to eliminate the impact of differences in inflorescence numbers among individuals on the analysis, the time-series data of individual floral abundance were first standardized. This was achieved by dividing the daily floral abundance by the total floral abundance of that individual, scaling the standardized floral abundance (*FA_s_*) between 0 and 1:

(1) $${FA}_{s}=\frac{{FA}_{i}}{\sum_{i=1}{FA}_{i}}$$

where *FA_s_* is the standardized floral abundance for a specific species, and *FA_i_* is the actual floral abundance of that species on monitoring date *i*.

Considering that the time-series data of floral abundance for alpine grassland plants typically exhibit a positively skewed distribution [47], skewness was calculated based on the floral abundance distribution data of each individual. A skewness greater than 0 indicates a right-skewed distribution, implying that the time from the onset of flowering to peak flowering is shorter than the time from peak flowering to the end of flowering. A skewness equal to 0 indicates a symmetrical distribution, while a value less than 0 indicates a left-skewed distribution. Building on this, a four-parameter Gumbel probability density function was used to smoothly fit the standardized floral abundance series:

(2) $$y=y_{0}+A\;exp\left[-exp\left[-\left(\frac{{x-x}_{c}}{\omega }\right)\right]-\left(\frac{{x-x}_{c}}{\omega }\right)+1\right]$$

where *y*_0_ is the vertical offset of the curve, *A* is the amplitude between *y*_0_ and the highest point of the curve, *x_c_* is the independent variable value corresponding to the curve’s peak representing the position parameter, and *ω* is the curve width representing the scale parameter.

The F-test was utilized to determine the fitting significance (*p* < 0.05), and R^2^ was used to evaluate the goodness of fit. For well-fitted curves, the date corresponding to the curve’s peak was defined as the peak flowering date (PFD). The dates when the standardized floral abundance first reached 0.05 and declined to 0.05 on either side of the curve were defined as the first flowering date (FFD) and the last flowering date (LFD), respectively. Flowering duration (FD) was calculated as the time difference between LFD and FFD.

To comparatively analyze phenological characteristics across hierarchical levels, phenological characteristics were further analyzed at the population, functional group, and community levels. At the population level, the mean floral abundance of all individuals of each species on each date was used as its time-series distribution of floral abundance, and phenological parameters were then extracted using the same method applied at the individual level. Based on the three population-level parameters—FFD, PFD, and LFD—all plants were categorized into three functional groups (early-, mid-, and late-flowering) using K-means clustering. Time-series distribution data of flower abundance at the functional group and community levels were obtained by summing the flower abundance data of all individuals within the group or community by date. Subsequently, phenological characteristics for each functional group and the community were derived through the identical standardization, curve-fitting, and threshold framework.

To elucidate the flowering modes of different plant species and groups, the flowering synchrony index (*S_i_*) among individuals within each species was calculated [13]:

(3) $$S_{i}=\frac{1}{n-1}\left(\frac{1}{{FD}_{i}}\right)\sum_{j=1}^{n}e_{j\neq i}$$

where *e_j_* represents the overlap time of the flowering periods between individuals *i* and *j*, *FD_i_* is the flowering duration of individual *i*, and *n* is the total number of individuals within the species.

*S_i_* ranges from 0 to 1, representing varying degrees of flowering synchrony between individual *i* and other individuals. A value of “0” indicates no overlap, whereas “1” indicates complete overlap. Subsequently, the mean of the flowering synchrony indices of all individuals within a species, all individuals within a functional group, and all individuals within the community were used as the flowering synchrony indices for the species, group, and community levels, respectively.

### 4.5. Statistical Analysis

Due to the predominantly skewed distribution of intraspecific individual phenological parameters, medians and interquartile range (IQRs) were used for description descriptive statistics after determining the specific individual values. Correlations among phenological parameters at the individual level were tested using Spearman’s rank correlation coefficients. At the population level, correlations among phenological parameters (FFD, PFD, LFD, FD, skewness, and synchrony) were tested using Pearson correlation coefficients. Differences in phenological parameters among functional groups were analyzed using the Kruskal-Wallis non-parametric analysis of variance, followed by Dunn’s test for pairwise comparisons. All statistical analyses were conducted in R version 4.5.0, with the significance level set at *p* = 0.05.

## 5. Conclusions

This study systematically delineates the hierarchical flowering phenology of an alpine entomophilous plant community. We found that sympatric plants partition the limited reproductive season through temporal niche differentiation, forming early-, mid-, and late-flowering functional groups that collectively drive a bimodal community floral display. Notably, these functional groups exhibit divergent flowering strategies, ranging from mass-flowering in early-flowering species to extended, steady-flowering in mid-flowering species. Concurrently, high intraspecific variation in individual phenological parameters was observed throughout the season. Community phenology is therefore not driven by a singular reproductive strategy but represents a complex composite of diverse temporal patterns. In the context of alpine environments, these varying inter- and intraspecific phenological traits likely reflect complex ecological trade-offs to balance limited time for reproduction against variable pollination opportunities. Ultimately, this highlights the necessity of multi-scale phenological monitoring for accurately understanding community structure and predicting its potential shifts under future climate change.

## Figures and Tables

**Table 1:** Median and interquartile range of flowering phenological parameters of intra-specific individuals.

Family	Species	FFD	PFD	LFD	FD (d)	Si
Ranunculaceae	*Pulsatilla turczaninovii*	159 (10)	164 (6)	171 (3)	8 (5)	0.60 (0.15)
Rosaceae	*Sibbaldianthe adpressa*	159	160	162	3	-
Iridaceae	*Iris tenuifolia*	161 (7)	162 (7)	164 (7)	4 (1)	0.15 (0.32)
Violaceae	*Viola betonicifolia*	164 (21)	165 (18)	168 (16)	5 (6)	0.10 (0.30)
Ranunculaceae	*Trollius farreri*	167 (6)	172 (2)	184 (6)	14 (6)	0.76 (0.12)
Ranunculaceae	*Anemone subpinnata*	168 (8)	172 (7)	180 (10)	11 (14)	0.46 (0.22)
Rosaceae	*Potentilla bifurca*	172 (9)	178 (6)	189 (7)	16 (13)	0.63 (0.10)
Brassicaceae	*Braya humilis*	174 (29)	189 (33)	210 (46)	33 (14)	0.37 (0.20)
Mazaceae	*Lancea tibetica*	175	180	185	10	-
Fabaceae	*Oxytropis bicolor*	176 (8)	180 (7)	189 (7)	10 (8)	0.54 (0.18)
Thymelaeaceae	*Stellera chamaejasme*	177 (6)	185 (3)	192 (3)	15 (8)	0.73 (0.10)
Rosaceae	*Potentilla multicaulis*	177 (43)	188 (32)	200 (30)	5 (21)	0.36 (0.36)
Rosaceae	*Potentilla saundersiana*	180 (6)	185 (6)	192 (11)	13 (10)	0.56 (0.22)
Fabaceae	*Tibetia himalaica*	182 (1)	182 (3)	186 (8)	3 (8)	0.58 (0.34)
Caryophyllaceae	*Stellaria palustris*	182 (16)	190 (16)	202 (14)	4 (18)	0.50 (0.25)
Fabaceae	*Oxytropis coerulea*	182 (8)	187 (6)	192 (12)	7 (6)	0.47 (0.23)
Ranunculaceae	*Ranunculus tanguticus*	182 (7)	182 (6)	191 (6)	6 (4)	0.41 (0.15)
Rosaceae	*Potentilla sischanensis*	188 (23)	192 (20)	202 (18)	5 (11)	0.27 (0.11)
Gentianaceae	*Gentiana pseudoaquatica*	189 (50)	190 (50)	190 (50)	1 (0)	0.10 (0.03)
Plantaginace	*Veronica ciliata*	198 (3)	200 (2)	202 (4)	6 (1)	0.67 (0.26)
Fabaceae	*Oxytropis ochrocephala*	198 (17)	202 (11)	212 (6)	12 (6)	0.57 (0.41)
Polygonaceae	*Bistorta vivipara*	206 (2)	214 (2)	230 (3)	25 (6)	0.97 (0.15)
Fabaceae	*Astragalus laxmannii*	206 (20)	211 (15)	226 (8)	18 (18)	0.56 (0.17)
Fabaceae	*Medicago archiducis-nicolai*	208 (17)	214 (14)	226 (7)	14 (14)	0.51 (0.17)
Asteraceae	*Taraxacum mongolicum*	209 (26)	210 (26)	210 (26)	1 (0)	0.10 (0.25)
Caryophyllaceae	*Silene repens*	210 (1)	214 (2)	222 (12)	12 (14)	0.65 (0.47)
Amaryllidaceae	*Allium przewalskianum*	214	219	227	13	-
Asteraceae	*Anaphalis lactea*	214 (3)	219 (7)	231 (9)	18 (7)	0.74 (0.20)
Fabaceae	*Oxytropis kansuensis*	218 (8)	226 (6)	242 (11)	17 (20)	0.64 (0.18)
Gentianaceae	*Gentiana dahurica*	219 (4)	224 (5)	234 (6)	14 (9)	0.66 (0.25)
Fabaceae	*Gueldenstaedtia verna*	220 (4)	225 (6)	232 (12)	11 (20)	0.60 (0.22)
Asteraceae	*Saussurea hieracioides*	220 (13)	223 (17)	226 (12)	6 (2)	0.57 (0.46)
Asteraceae	*Aster lautureanus*	221 (3)	236 (2)	251 (3)	29 (3)	0.95 (0.08)
Gentianaceae	*Gentiana pudica*	226 (1)	232 (5)	245 (7)	20 (7)	0.90 (0.14)
Asteraceae	*Aster souliei*	230 (50)	232 (58)	238 (66)	8 (17)	0.30 (0.30)
Campanulaceae	*Adenophora stenanthina*	235	238	240	5	-

The data shown in the table are the median (interquartile range) of flowering parameters of intraspecific individuals. FFD: first flowering date; PFD: peak flowering date; LFD: last flowering date; FD: flowering duration; Si: flowering synchrony index among individuals within each species. Taxa are ordered by FFD rather than taxonomically, to illustrate the temporal sequence of species in the community.

## Data Availability

Dataset available on request from the authors: The raw data supporting the conclusions of this article will be made available by the authors on request.
